# KIR3DS1/HLA-B Bw4-80Ile Genotype Is Correlated with the IFN-α Therapy Response in hepatitis B e antigen-Positive Chronic Hepatitis B

**DOI:** 10.3389/fimmu.2017.01285

**Published:** 2017-10-11

**Authors:** Wenting Li, Xiaokun Shen, Binqing Fu, Chuang Guo, Yanyan Liu, Ying Ye, Rui Sun, Jiabin Li, Zhigang Tian, Haiming Wei

**Affiliations:** ^1^The CAS Key Laboratory of Innate Immunity and Chronic Disease, School of Life Sciences and Medical Center, Institute of Immunology, University of Science and Technology of China, Hefei, China; ^2^Hefei National Laboratory for Physical Sciences at Microscale, University of Science and Technology of China, Hefei, China; ^3^Department of Infectious Diseases, the First Affiliated Hospital of Anhui Medical University, Hefei, China

**Keywords:** killer-cell immunoglobulin-like receptors/human leukocyte antigen, hepatitis B virus, therapy, interferon alpha, sustained response, genotype

## Abstract

To date, several on-treatment-level virological and serological indices that may predict the response to interferon alpha (IFN-α) have been reported. However, no effective predictors, such as drug–response genes, that can be detected before administration of anti-hepatitis B virus (HBV) therapy with IFN-α, have been found. In the diverse range of chronic viral infection, genes that affect human immunity play important roles in understanding host and viral co-evolution. Killer-cell immunoglobulin-like receptors (KIRs), which are highly polymorphic at the allele and haplotype levels, participate in the antiviral function of natural killer (NK) cells *via* fine-tuning inhibition and activation of NK-cell responses that occur when the NK cells interact with human leukocyte antigen (HLA) class I molecules on target cells. For each individual, the pairing of KIR and HLA ligand is genetically determined. To investigate whether a particular KIR and HLA repertoire influences the risk of HBV infection and response to IFN-α treatment for chronic hepatitis B (CHB), we genotyped the KIRs and HLA ligands of 119 hepatitis B e antigen (HBeAg)-positive CHB patients. These patients included 43 patients who achieved sustained response (SR) induced by IFN-α treatment for 48 weeks, 76 patients who achieved no response (NR), and 96 healthy subjects as controls. SR was defined as HBeAg loss with HBV DNA < 2,000 IU/ml and alanine aminotransferase normalization at 24 weeks posttreatment (week 72). In this study, we showed that activating KIR genes were less prevalent in Han Chinese, especially in Han Chinese with CHB, than in Caucasians. Furthermore, the KIR3DS1 gene, in combination with HLA-B Bw4-80Ile, strongly influenced the therapeutic outcomes for CHB patients who were treated with IFN-α. The frequency of the combination of genes encoding KIR3DS1 and HLA-B Bw4-80Ile was higher in patients who had a sustained treatment response than in patients who had NR [35.3 versus 1.3%; odds ratio (OR) = 19.85; *P* = 0.0008]. Activating KIR3DS1 and HLA-B Bw4-80Ile synergistically predicted SR to IFN-α for HBeAg-positive CHB patients. Genotyping for the KIR3DS1 gene and the HLA-B Bw4-80Ile allele might help physicians choose the optimal candidates for anti-HBV treatment with IFN-α.

## Introduction

Hepatitis B virus (HBV) infection, with 248 million chronic hepatitis B (CHB) surface antigen (HBsAg) carriers, is a worldwide health concern (1). Patients with persistent hepatitis B infection carry a high risk of developing cirrhosis, liver failure, and hepatocellular carcinoma (2). Factors that affect the resolution or persistence of infection include age at infection, immunodeficiency status, gender, and host genetic variation (3, 4). Hepatitis B is endemic to Asia and parts of Africa but is much less prevalent in North America and Europe (5, 6). Whether this chronic infection is associated with genetic differences among different populations remains to be studied. Currently, CHB treatment includes several common strategies: (1) suppression of HBV replication through treatment with nucleoside or nucleotide analogs (NAs), such as lamivudine, adefovir, telbivudine, entecavir, and/or tenofovir; (2) immunomodulation with interferon alpha (IFN-α); or a combination of both approaches (7, 8). Among the current therapies for CHB, IFN-α is still the most effective drug with the highest off-treatment sustained response (SR) rate after a 48-week course of therapy and with relatively low risk of posttreatment relapse (7, 8). However, treatment with IFN-α is often associated with side effects, such as flu-like symptoms, thrombocytopenia, psychiatric disorders (9), and, most importantly, a limited SR rate (7). Thus, new indicators for the prediction of SR to IFN-α are urgently needed.

Increasing evidence has supported a role for natural killer (NK) cells, which are key components of the innate immune system, in the response to chronic HBV infection. NK cells, which constitute the predominant lymphocyte subpopulation in the liver, have the capacity to exert an antiviral function early in infection, thereby contributing to early viral containment. However, as chronic infection develops, NK cells become significantly less activated and are partially functionally tolerant under the influence of the tolerogenic liver microenvironment (10, 11). NK cell function is determined by the balance of activating and inhibitory NK cell receptor signals. In recent years, we have reported that reduced expression of the activating receptor NKG2D dampened NK cell function in immune-tolerant phase HBV patients. Additionally, the inhibitory receptor NKG2A, which is the receptor for human leukocyte antigen (HLA)-E, impairs NK cell function in immune-active patients, and NKG2A blocking can upregulate the activity of NK cells and result in the clearance of HBV infection (12, 13). Killer-cell immunoglobulin-like receptors (KIRs), another large group of NK cell receptors, can recognize specific HLA molecules expressed on target cells and exert an immune surveillance function. KIR and HLA are encoded on separate chromosomes by genes with highly allelic polymorphisms, and their variants and combinations of these variants are strongly associated with resistance or susceptibility to infectious diseases (14). Based on the types and numbers of KIR genes, KIR haplotypes can be classified into two broad classes consisting of the A and B haplotypes (15). Only several defined HLA alleles have been confirmed to be ligands specifically interacting with KIRs. A weak inhibitory KIR and HLA interaction that can be easily overcome results in NK cell activation, which has been confirmed to occur in hepatitis C virus (HCV) resolution and in the better treatment response to chronic hepatitis C infection (16, 17). A recent study suggested that KIR2DL3 and KIR ligands HLA-A Bw4 or HLA-C2 were related to spontaneous resolution of the primary HBV infection (18). However, no candidate gene has been identified to predict the outcome of anti-HBV treatment. Therefore, it is urgent to investigate whether KIR/HLA plays a role in IFN-α therapy for chronic HBV infection.

The aim of the current study was to test the hypothesis that KIR and HLA genes might separately or in combination affect HBV infection resolution or predict responses to anti-HBV therapy.

## Materials and Methods

### Patient Characteristics

A total of 119 hepatitis B e antigen (HBeAg)-positive CHB patients who had received anti- HBV treatment for 48 weeks and 96 healthy subjects were enrolled in this study. CHB patients were provided written informed consent, and this study was approved by the Ethics Committee of the First Affiliated Hospital of Anhui Medical University (Grant No. K2010003). The clinical research was registered in the Chinese Clinical Trial Registry (registration number: ChiCTR-TRC-12002226). All of the CHB patients in this study ranged in age from 18 to 65 years with high HBV DNA levels (>20,000 IU/ml) and alanine aminotransferase (ALT) levels between 2 and 10 times the upper limit of normal (40 IU/l). Exclusion criteria included coinfection with HCV, hepatitis delta virus, human immunodeficiency virus (HIV), or any other viral infectious disease. Two treatment strategies were performed among the 119 CHB patients. Twenty-seven CHB patients received IFN-α monotherapy, and the other 92 CHB patients received combination therapy with IFN-α and NAs for 48 weeks. IFN-α was administered by intramuscular injection (PEG-IFN-α-2b, 1.5 μg/kg/week), and NAs were given orally at the regular clinical dose for 48 weeks (ADV, 10 mg/day). SR to treatment was defined according to the European Association for the Study of the Liver Guidelines (19) as HBeAg < 1 COI, HBV DNA < 2,000 IU/ml and ALT < 40 IU/l when follow-up at 24 weeks posttreatment (week 72) in both of the treatment groups. Patients were considered to have no response (NR) to therapy if they did not meet all of the criteria above. The healthy control group consisted of 96 blood donors without HBV infection. Blood samples from healthy subjects were processed from the Laboratory Centre of the First Affiliated Hospital of Anhui Medical University with approval from the Ethics Committee of the First Affiliated Hospital of Anhui Medical University.

### Measurement of Clinical Indicators

The serum samples for clinical indicator measurement were obtained from fresh whole blood collected in tube without anticoagulants. All of the clinical indicators were quantified in samples taken at baseline and during treatment period and follow-up periods. Sampling was performed at weeks 0, 4, 8, 12, 24, 36, 48, and 72 (week 0 was the time-point at which treatment began). HBV DNA quantification was measured using the Roche COBAS TaqMan HBV Test (F. Hoffman-La Roche Ltd., Basel, Switzerland) with a lower limit of detection <20 IU/ml. HBsAg was quantifed using the Roche Elecsys HBsAg II quant assay (Roche Diagnostics, China). HBeAg was measured using a Roche Elecsys 2010 Immunoanalyzer (Roche Diagnostics, China). Serum ALT was detected at the time of sampling in accordance with standard procedures. HBV genotyping performed using polymerase chain reaction (PCR) assays and genotype-specific primers (20).

### KIR and HLA Genotyping

The peripheral blood samples for genotyping were collected in anticoagulative tube without strict time-point limitations over the treatment period. Peripheral blood mononuclear cells were obtained by removing the red blood cells (RBC) from the whole blood by using RBC Lysis Buffer (Biolegend) and stored in a −80°C freezer. The genomic DNA for genotyping was then extracted from peripheral blood mononuclear cells using the Wizard^®^ SV Genomic DNA Purification System (Promega, USA). Sixteen KIR genes (seven inhibitory receptors, including KIR2DL1, KIR2DL2, KIR2DL3, KIR2DL5, KIR3DL1, KIR3DL2, and KIR3DL3 and seven activating receptors, including KIR2DL4, KIR2DS1, KIR2DS2, KIR2DS3, KIR2DS4, KIR2DS5, and KIR3DS1) plus two pseudogenes (KIR2DP1 and KIR3DP1) were analyzed. KIR genotyping was performed *via* PCR with sequence-specific primers (PCR-SSP) using a KIR-TYPE kit (BAG Health Care GmbH, Lich, Germany) or methods previously reported (21, 22). The KIR ligand groups, including HLA-C1, HLA-C2, and HLA-Bw4, were determined by PCR-SSP using either the Epitop-TYPE Kit (BAG Health Care GmbH, Lich, Germany) or a previously reported method (23).

### Statistical Analyses

Statistical comparisons between two groups were performed using the two-tailed Student’s *t*-test in GraphPad Prism 6.0 (GraphPad Software, Inc.). Values in the bar graphs are presented as means ± SEM. The chi-square test as calculated from a 2 × 2 contingency table was used to examine the differences in gene and genotype frequencies between the groups of subjects. The data were also analyzed using a stepwise multiple variable logistic regression model with SPSS (Version 16.0; SPSS for Windows). In this model, the KIR gene and HLA ligand were factors that were force entered into the model. The conditioning variables were entered into the model as stepwise terms. A backward elimination procedure was conducted to obtain the final set of significant covariates reported in Table 4. The chi-square test for trend was calculated using OpenEpi (Version 3.03a).

## Results

### Lower Frequencies of Activating KIR Genes in Han Chinese than in Caucasians

**Figure 1:**
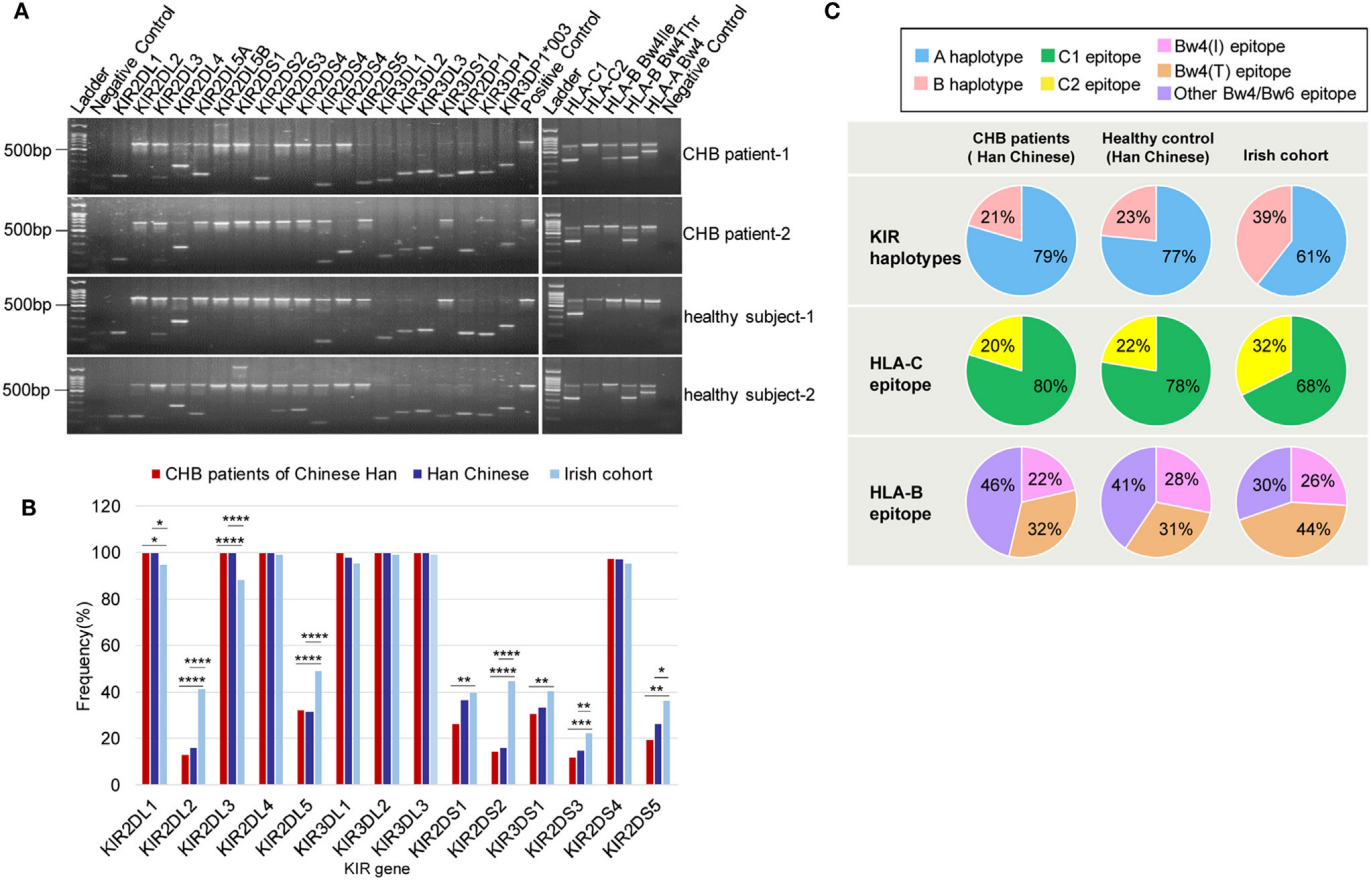
Killer-cell immunoglobulin-like receptor (KIR)/human leukocyte antigen (HLA) genes frequencies among Han Chinese chronic hepatitis B (CHB) patients, healthy Han Chinese subjects and the Irish cohorts. **(A)** Genotyping for KIRs and HLA ligand epitopes by using PCR-SSP. Positive control (659 bp), 2DL1 (142 bp), 2DL2 (142 bp), 2DL3 (135 bp), 2DL4 (252 bp), 2DL5A/B (163 bp/1640 bp), 2DS1 (143 bp), 2DS2 (204 bp), 2DS3 (212 bp), 2DS4 (99 bp/171 bp), 2DS4*008(171 bp), 2DS5 (108 bp), 3DL1 (136 bp), 3DL2 (180 bp), 3DL3 (191 bp), 3DS1 (147 bp), 2DP1 (168 bp), 3DP1 (157 bp), 3DP1*003 (157 bp/233 bp), HLA-C1 (326 bp), HLA-C2 (323 bp), HLA-B Bw4-80Thr (356 bp), HLA-B Bw4-80Ile (356 bp), and HLA-A Bw4 (392 bp). **(B)** Relative frequencies of inhibitory and activating KIR genes among Han Chinese CHB patients (red) (*N* = 119), healthy Han Chinese subjects (dark blue) (*N* = 125), and the Irish cohorts (light blue) (*N* = 322) (27). KIR gene frequencies are similar between the Han Chinese CHB patients and Han Chinese healthy subjects but differ according to ethnicity. **(C)** Pie charts compare the distribution of KIR haplotypes, and the HLA-C and HLA-B epitopes recognized by KIRs in Han Chinese CHB patients (*N* = 119), healthy Han Chinese subjects (*N* = 125), and the Irish cohorts (*N* = 322) (27). The B haplotype (pink) containing mostly activating KIR genes is less conserved than the A haplotype (blue). HLA-C1 (green), which is carried by HLA-C allotypes with asparagine at position 80 is the ligand for KIR2DL2/2DL3 and HLA-C2 (yellow), carried by HLA-C allotypes, with lysine at position 80, is the ligand for KIR2DL1/2DS1. HLA-B Bw4-80Ile (rose) and HLA-B Bw4-80Thr (brownish) are alleles of HLA-B Bw4. Bw4 and Bw6 (purple) are two different serological epitopes of the HLA-A and HLA-B allotypes, with arginine and lysine at position 83, respectively. Abbreviations: HLA, human leukocyte antigen; KIR, killer immunoglobulin-like receptor; CHB, chronic hepatitis B.

To study the correlation between particular KIR or HLA genes and chronic HBV infection, 119 CHB patients and 96 healthy subjects, all of whom were Han Chinese (Table S2 in Supplementary Material), were genotyped for 19 KIR genes and three major KIR ligands (HLA-C1, HLA-C2, and HL-Bw4) (Figure 1A). HLA-C1 is the ligand for KIR2DL2 and KIR2DL3, whereas HLA-C2 is the ligand for KIR2DL1 and KIR2DS1. In addition, KIR2DL2/L3 could also have a weaker interaction with HLA-C2 than HLA-C1 (24, 25). Moreover, whether KIR2DS2 recognition is independent of the HLA-C1 is controversial (26). The Bw4 epitope, which contains alleles that have an isoleucine at position 80 (Bw4-80Ile) or a threonine at this position (Bw4-80Thr) can interact with KIR3DL1, but interactions with KIR3DS1 are still unconfirmed. Univariate analysis revealed that, the frequencies of subjects who possessed the activating genes KIR2DS1 and KIR2DS5 were slightly lower in the CHB patient group than in the healthy subject group, but no significant differences between the two groups were noted (Figure 1B; Table S1 in Supplementary Material). Notably, except for KIR2DS4, which is present in both the A and B haplotypes, the frequencies of subjects with activating KIRs unique to the B haplotype (KIR2DS1, KIR2DS2, KIR2DS3, KIR2DS5, and KIR3DS1) were much lower, in both Han Chinese CHB patients and healthy subjects than in Caucasians [Irish cohorts (27) and United States (US) Caucasians (28)] (Figure 1B; Table S1 in Supplementary Material). Incidentally, the Caucasian data were obtained from the general population categorized only by ethnicity and region, but was not restricted to the healthy subjects. This finding suggested that a deficiency in activating KIRs (the AA haplotype) in Han Chinese likely results in a greater susceptibility to developing CHB. Individuals who possessed the inhibitory molecules KIR2DL2 and KIR2DL5, both of which are specific to the B haplotype, were less prevalent among Han Chinese than among Irish and US Caucasians cohorts (27, 28) (Figure 1B; Table S1 in Supplementary Material).

In addition, although there were no differences in the KIR haplotype and the HLA-C allele frequencies between CHB patients and healthy subjects of Han Chinese, both the KIR B haplotype and the HLA-C2 epitope were more frequent in the Caucasian population [Irish cohorts (27)] than in Han Chinese CHB patients and healthy Han Chinese subjects (Figure 1C). However, the Bw4 epitope had similar distribution among the different groups that were studied (Figure 1C). These data indicated that Han Chinese and Caucasians have different carrier rates of certain KIR or HLA ligand genes.

### KIRs/HLA and HBV Infection

Because KIRs interact with specific HLA class I molecules, we considered the KIR/HLA gene combinations as either risk or protective factors. To investigate whether there was a difference between CHB patients and healthy subjects possessing a specific KIR-HLA genes combination, we further compared all of the KIR2D/HLA-C and KIR3D/HLA-B Bw4 combinations (KIR2DL1 + HLA-C1, KIR2DL2 + HLA-C2, KIR2DL3 + HLA-C2, KIR2DS1 + HLA-C2, KIR2DS2 + HLA-C1, KIR2DL2/2DL2 + HLA-C1C1, KIR2DL2/2DL3 + HLA-C1C1, KIR2DL3/2DL3 + HLA-C1C1, KIR2DL3/2DL3 + HLA-C1C2, KIR2DL3/2DL3 + HLA-C2C2, KIR3DL1 + HLA-B Bw4-80Ile, KIR3DL1 + HLA-B Bw4-80Thr, KIR3DS1 + HLA-B Bw4-80Ile, and KIR3DS1 + HLA-B Bw4-80Thr). The frequency of KIR2DS1 + HLA-C1C1 genotype was slightly higher in the CHB patients than the healthy subjects (5 versus 0%, *P* = 0.03), however, the frequency of KIR2DS1 + HLA-C1 genotype was not different in the two groups (8.4 versus 12.5%, OR = 0.64, *P* = 0.32). The KIR2DL2/2DL3 + HLA-C1C1 genotype, which has previously been implicated in spontaneous clearance of HCV virus (16), was enriched in the CHB patient group than the healthy subject group (16.8 versus 7.3%; *P* = 0.04). Moreover, we found no KIR2DL2/HLA-C1 homozygous genotypes in either group due to its rare frequency in the Han Chinese population. No other differences were found in relation to HBV infection (Table 1).

**Table 1:** Frequencies of HLA and KIR-HLA combinations among CHB patients and healthy subjects.

Genetic factor	CHB patients	Healthy subjects	OR^a^	95% confidence interval	*P*^b^
	*N* (%)	*N* (%)			
	*N* = 119	*N* = 96			
HLA-C1C1	76 (63.9)	57 (59.4)	1.21	0.70–2.10	0.50
HLA-C1C2	38 (31.9)	36 (37.5)	0.78	0.44–1.38	0.39
HLA-C2C2	5 (4.2)	3 (3.1)	1.36	0.32–5.84	0.96
2DL2 + HLA-C1	19 (15.3)	14 (14.6)	1.11	0.53–2.35	0.91
2DL3 + HLA-C1	114 (95.8)	92 (95.8)	2.07	0.66–6.56	1.00
2DS2 + HLA-C1	15 (12.6)	14 (14.6)	0.84	0.39–1.85	0.67
2DL2 + HLA-C1C1	14 (11.8)	7 (7.3)	1.70	0.66–4.38	0.27
2DL3 + HLA-C1C1	76 (63.9)	57 (59.4)	1.21	0.70–2.10	0.50
2DS2 + HLA-C1C1	13 (10.9)	7 (7.3)	1.84	0.67–5.04	0.36
2DL1 + HLA-C2	39 (32.8)	39 (40.6)	0.71	0.41–1.25	0.50
2DS1 + HLA-C2	10 (8.4)	12 (12.5)	0.64	0.26–1.56	0.32
2DL1 + HLA-C2C2	3 (2.5)	3 (3.1)	0.80	0.16–4.07	1.00
**2DS1 + HLA-C2C2**	6 (5.0)	0 (0)	–	–	**0.03**
2DL2/2DL2 + C1C1	0 (0.0)	0 (0)	–	–	1.00
**2DL2/2DL3 + C1C1**	20 (16.8)	7 (7.3)	2.57	1.04–6.36	**0.04**
2DL3/2DL3 + C1C1	56 (47.1)	50 (52.1)	0.82	0.48–1.40	0.46
2DL3/2DL3 + C1C2	27 (22.7)	29 (30.2)	1.05	0.55–2.00	0.21
2DL3/2DL3 + C2C2	2 (1.7)	2 (2.08)	0.53	0.09–3.24	1.00
B Bw4-80T	44 (37.0)	33 (34.4)	1.17	0.67–2.06	0.69
B Bw4-80I	29 (24.4)	30 (31.3)	0.78	0.43–1.44	0.26
HLA-B Bw4-80TT	36 (30.3)	27 (28.1)	1.17	0.64–2.12	0.73
HLA-B Bw4-80TI	6 (5.0)	6 (6.3)	0.80	0.25–2.55	0.70
HLA-B Bw4-80II	23 (19.3)	24 (25.0)	0.81	0.42–1.56	0.32
3DL1 + B Bw4-80T	44 (37.0)	33 (34.4)	1.17	0.67–2.06	0.70
3DL1 + B Bw4TT	36 (30.3)	27 (28.1)	1.17	0.64–2.12	0.73
3DL1 + B Bw4-80I	29 (24.4)	30 (31.3)	0.78	0.43–1.44	0.26
3DL1 + B Bw4-80II	23 (19.3)	24 (25.0)	0.81	0.42–1.56	0.32
3DS1 + B Bw4-80I	10 (8.4)	11 (11.5)	0.89	0.35–2.28	0.45
3DS1 + B Bw4-80II	8 (6.7)	9 (9.4)	3.39	0.70–16.34	0.47

*KIR, killer immunoglobulin-like receptor; HLA, human leukocyte antigen; CHB, chronic hepatitis B; OR, odds ratio*.

*^a^OR > 1 indicates a protective association with response to treatment*.

*^b^P values were calculated by using a Chi-square test from a 2 × 2 contingency table*.

*Bold font indicates statistical significance (*P* < 0.05)*.

### The Activating KIR Gene KIR3DS1 Correlates with the Therapeutic Response in CHB Patients

We divided the 119 CHB patients enrolled in this study into two groups: based on curative effects at 24 weeks posttreatment (week 72): (1) SR group and (2) NR group, according to their curative effects. Forty-three out of the 119 CHB patients achieved SR while the other 76 CHB patients had NR after receiving 48 weeks of IFN-α monotherapy or IFN-α plus NA combination treatment. To identify whether KIR and the HLA ligand contributed to the anti-HBV treatment response, we compared a particular KIR or HLA genes between the SR and NR groups and found that the AB haplotype that includes at least one activating KIR had higher frequency in the SR patients group than in the NR patient group. The frequencies of only two of 19 KIR genes analyzed (KIR3DS1 and KIR2DL5, both of which are located in the AB haplotype and are physically linked to each other) showed significant differences between the SR and NR groups, being more prevalent in the SR group (51.2 and 44.2%, respectively) than the NR group (18.4 and 25.0%, respectively) [odds ratio (OR) = 4.64, *P* = 0.0002; OR = 2.38, *P* = 0.03]. This finding also supports a role for KIR3DS1 in the defense against HBV (Table 2). However, neither the frequency of the subjects possessing HLA-B Bw4Thr allele nor that of the subjects possessing the HLA-B Bw4Ile allele, which encodes a more effective ligand for KIR3DL1 than the Bw4-80Thr allele, differed between the SR and NR groups (OR = 1.96, *P* = 0.12; OR = 1.02, *P* = 0.97) (Table 2). The frequency of subjects possessing the Bw6 serotype was lower in the SR group (76.7%) than in the NR group (90.8%) (OR = 0.33, *P* = 0.04) (Table 2). The above analysis indicates that the KIR3DS1 gene may affect therapeutic responses to chronic HBV infection.

**Table 2:** Correlation of single KIR or HLA gene with CHB patients therapeutic response.

Genetic factor	SR	NR	OR^a^	95% confidence interval	*P*^b^
	*N* (%)	*N* (%)			
	*N* = 43	*N* = 76			
KIR2DL1	43 (100.0)	76 (100.0)	–	–	–
KIR2DL2	7 (16.3)	11 (14.5)	1.15	0.41–3.22	0.79
KIR2DL3	43 (100.0)	76 (100.0)	–	–	–
KIR2DL4	43 (100.0)	76 (100.0)	–	–	–
**KIR2DL5**	19 (44.2)	19 (25.0)	2.38	1.07–5.26	**0.03**
KIR3DL1	43 (100.0)	76 (100.0)	–	–	–
KIR3DL2	43 (100.0)	76 (100.0)	–	–	–
KIR3DL3	43 (100.0)	76 (100.0)	–	–	–
KIR2DS1	15 (34.9)	16 (21.1)	2.01	0.87–4.63	0.10
KIR2DS2	6 (14.0)	11 (14.5)	0.96	0.33–2.80	0.94
**KIR3DS1**	22 (51.2)	14 (18.4)	4.64	2.02–10.67	**0.0002**
KIR2DS3	8 (18.6)	6 (7.9)	2.67	0.86–8.29	0.08
KIR2DS4	42 (97.7)	74 (97.4)	1.14	0.10–12.90	1.00
KIR2DS5	10 (23.3)	13 (17.1)	1.47	0.58–3.71	0.41
B Bw4	28 (65.1)	42 (55.3)	1.51	0.70–3.27	0.30
B Bw4-80Ile	14 (32.6)	15 (19.7)	1.96	0.84–4.60	0.12
B Bw4-80Thr	16 (37.2)	28 (36.8)	1.02	0.47–2.20	0.97
**Bw6**	33 (76.7)	69 (90.8)	0.33	0.12–0.96	**0.04**
HLA-C1	40 (93.0)	74 (97.4)	0.36	0.06–2.25	0.51
HLA-C2	18 (41.9)	25 (32.9)	1.47	0.68–3.18	0.33
**AA haplotype**	18 (41.8)	51 (67.1)	0.35	0.16–0.76	**0.007**
**AB haplotype**	25 (58.2)	25 (32.9)	2.83	1.31–6.13	**0.007**
BB haplotype	0 (0.0)	0 (0.0)	–	–	–

*KIR, killer immunoglobulin-like receptor; HLA, human leukocyte antigen; CHB, chronic hepatitis B; OR, odds ratio; SR, sustained response; NR, no response*.

*^a^OR > 1 indicates a protective association with response to treatment*.

*^b^P values were calculated by using the chi-square test from a 2 × 2 contingency table*.

*Marginal statistical significance (*P* < 0.1)*.

*Bold font indicates statistical significance (*P* < 0.05)*.

### The KIR3DS1/HLA-B Bw4-80Ile Combination is Significantly Associated with a Stronger Therapeutic Response for CHB Patients

Because the interaction between KIR and its HLA ligand determines the transmission of the KIR downstream signal, we further assessed whether the KIR/HLA pair correlated with the response to therapy in CHB infection. Notably, comparison of the frequencies between CHB patients with the KIR3DS1 and HLA-B Bw4-80Ile combination genotype in the SR group (20.9%) and NR group (1.3%) (OR = 19.85, *P* = 0.0008) showed that this genotype contributed to the response to IFN-α therapy in HBeAg-positive chronic HBV infection (Table 3). In addition, the frequency of the KIR2DS1 and HLA-C2C2 combination was also higher in the SR group (11.6%) than in the NR group (1.3%) (OR = 9.87, *P* = 0.04), while the KIR2DL3/HLA-C1 homozygous genotype was less prevalent in the SR group (32.6%) than in the NR group (55.3%) (OR = 0.39, *P* = 0.02) (Table 3).

**Table 3:** Distribution of HLA and KIR-HLA combinations among CHB patients with a SR or NR to therapy.

Genetic factor	SR	NR	OR^a^	95% CI	*P*^b^
	*N* (%)	*N* (%)			
	*N* = 43	*N* = 76			
HLA-C1C1	25 (58.1)	51 (67.1)	0.68	0.31–1.47	0.33
HLA-C1C2	15 (34.9)	23 (30.3)	1.23	0.56–2.74	0.60
HLA-C2C2	3 (7.0)	2 (2.6)	2.78	0.45–17.30	0.51
2DL2 + HLA-C1	7 (16.3)	11 (14.5)	1.28	0.45–3.66	0.79
2DL3 + HLA-C1	40 (93.0)	74 (97.4)	0.36	0.06–2.25	0.51
2DS2 + HLA-C1	5 (11.6)	11 (14.5)	0.78	0.25–2.41	0.81
2DL2 + HLA-C1C1	5 (11.6)	9 (11.8)	1.12	0.34–3.66	0.97
2DL3 + HLA-C1C1	25 (58.1)	51 (67.1)	0.68	0.31–1.47	0.33
2DS2 + HLA-C1C1	4 (9.3)	9 (11.8)	2.50	0.53–11.72	0.90
2DL1 + HLA-C2	18 (41.9)	25 (32.9)	1.47	0.68–3.18	0.33
2DS1 + HLA-C2	5 (11.6)	5 (6.6)	1.54	0.44–5.36	0.54
2DL1 + HLA-C2C2	2 (4.7)	2 (2.6)	1.80	0.25–13.29	0.61
**2DS1 + HLA-C2C2**	5 (11.6)	1 (1.3)	9.87	1.11–87.50	**0.04**
2DL2/2DL2 + C1C1	0 (0.0)	0 (0.0)	–	–	–
2DL2/2DL3 + C1C1	5 (11.6)	9 (11.8)	0.98	0.31–3.14	0.97
2DL2/2DL3 + C1C2	1 (2.3)	1 (1.3)	1.79	0.11–29.29	1.00
2DL2/2DL3 + C2C2	1 (2.3)	1 (1.3)	1.79	0.11–29.29	1.00
**2DL3/2DL3 + C1C1**	14 (32.6)	42 (55.3)	0.39	0.18–0.85	**0.02**
2DL3/2DL3 + C1C2	14 (32.6)	22 (28.9)	1.18	0.53–2.66	0.68
2DL3/2DL3 + C2C2	2 (4.7)	1 (1.3)	3.66	0.32–41.58	0.61
HLA-B Bw4-80TT	10 (23.3)	26 (34.2)	0.58	0.25–1.37	0.21
HLA-B Bw4-80TI	4 (9.3)	2 (2.6)	3.79	0.67–21.65	0.25
HLA-B Bw4-80II	10 (23.3)	13 (17.1)	1.62	0.63–4.13	0.41
3DL1 + B Bw4-80T	16 (37.2)	28 (36.8)	0.96	0.44–2.08	0.97
3DL1 + B Bw4-80TT	10 (23.3)	26 (34. 2)	0.58	0.25–1.37	0.21
3DL1 + B Bw4-80IT	4 (9.3)	2 (2.6)	3.79	0.67–21.65	0.25
3DL1 + B Bw4-80I	14 (32.6)	15 (19.7)	2.14	0.90–5.06	0.12
3DL1 + B Bw4-80II	10 (23.3)	13 (17.1)	1.62	0.63–4.13	0.41
3DS1 + B Bw4-80TT	4 (9.3)	7 (9.2)	1.01	0.28–3.67	1.00
3DS1 + B Bw4-80IT	2 (4.7)	0 (0.0)	–	–	0.13
**3DS1 + B Bw4-80I**	9 (20.9)	1 (1.3)	19.85	2.42–163.00	**0.0008**
**3DS1 + B Bw4-80II**	7 (16.3)	1 (1.3)	14.58	1.73–123.05	**0.006**

*KIR, killer immunoglobulin-like receptor; HLA, human leukocyte antigen; CHB, chronic hepatitis B; OR, odds ratio SR, sustained response; NR, no response; CI, confidence interval*.

*^a^OR > 1 indicates a protective association with response to treatment*.

*^b^*P* values were calculated using a Chi-square test from a 2 × 2 contingency table. statistical significance (*P* < 0.05)*.

*Marginal statistical significance (*P* < 0.1)*.

*Bold font indicates statistical significance (*P* < 0.05)*.

**Figure 2:**
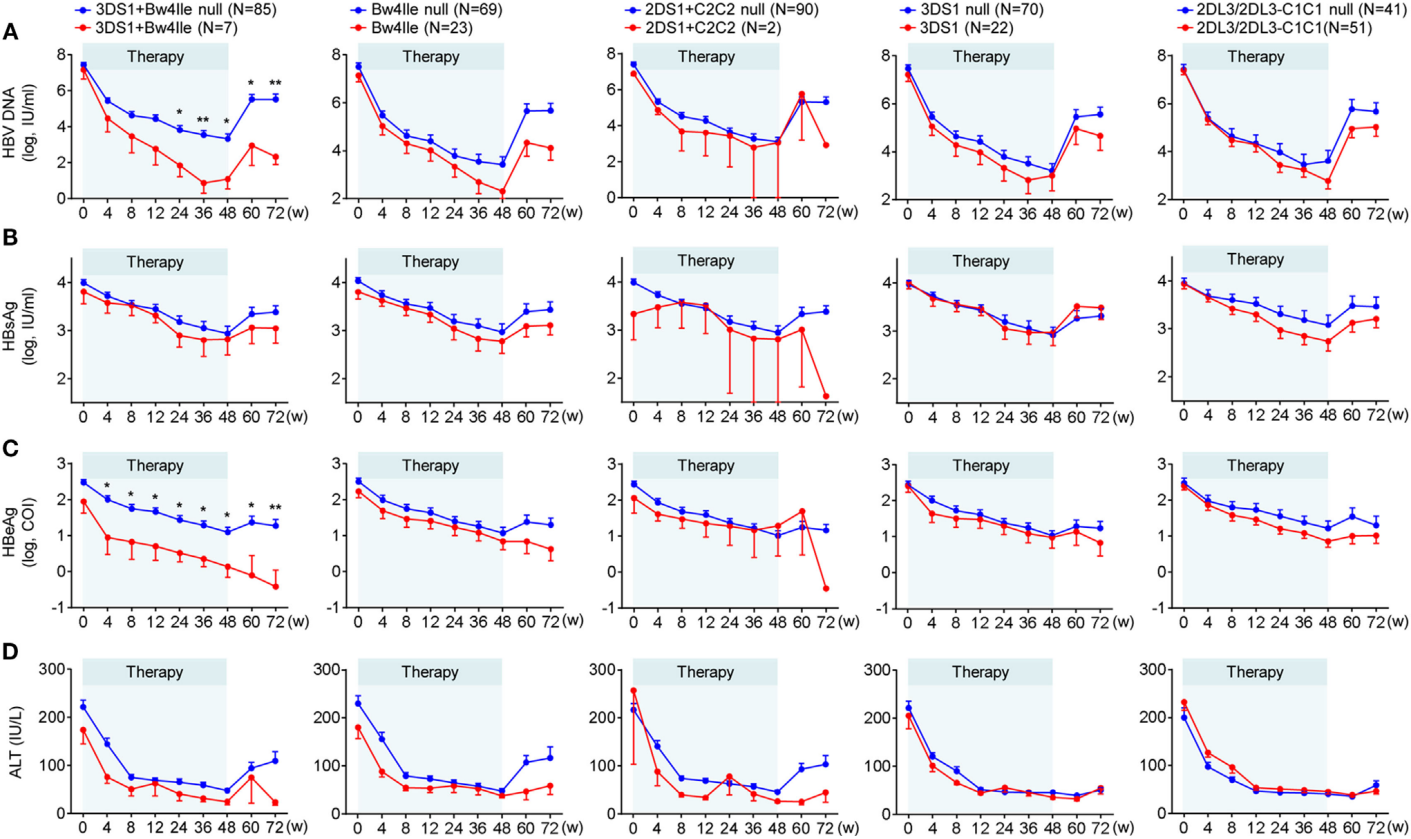
Chronic hepatitis B (CHB) patients with KIR3DS1/HLA-B Bw4-80Ile exhibited the best therapeutic response to CHB therapy. Five different killer-cell immunoglobulin-like receptor (KIR)/human leukocyte antigen (HLA) genetic factors, KIR2DS1/HLA-C2C2, KIR2DL3-2DL3/HLA-C1C1, KIR3DS1, HLA-B Bw4-80Ile, and KIR3DS1/HLA-B Bw4-80Ile, were analyzed independently. Mean changes in the serum hepatitis B virus (HBV) DNA **(A)**, HBsAg **(B)**, hepatitis B e antigen (HBeAg) **(C)** and ALT **(D)** level from baseline in patients (N = 92) with or without specific genotypes. Error bars, SEM *P < 0.05, **P < 0.01. Abbreviations: HBsAg, hepatitis B virus surface antigen; HBeAg, hepatitis B virus e antigen; ALT, alanine aminotransferase.

To investigate the influence of the KIR/HLA combination gene on chronic HBV infection in greater detail, we analyzed the dynamic variations of four clinical serological and virological indices (HBsAg, HBeAg, HBV DNA, and ALT) throughout the course of treatment. We found that the mean decrease in all four indices from pretreatment levels to week 72 was greater for patients possessing the KIR3DS1/HLA-B Bw4-80Ile combination genotype than for those without this combination; moreover, in particular, the levels of HBV DNA and HBeAg decreased considerably. However, a weaker correlation was found between decreases in these parameters and other genotypes (HLA-B Bw4-80Ile or KIR3DS1 alone, KIR2DS1/HLA-C2C2, and KIR2DL3/HLA-C1 homozygote) compared with the respective controls (Figures 2A–D), both during the course of treatment (0–48 weeks) and at 24 weeks posttreatment (week 72).

**Figure 3:**
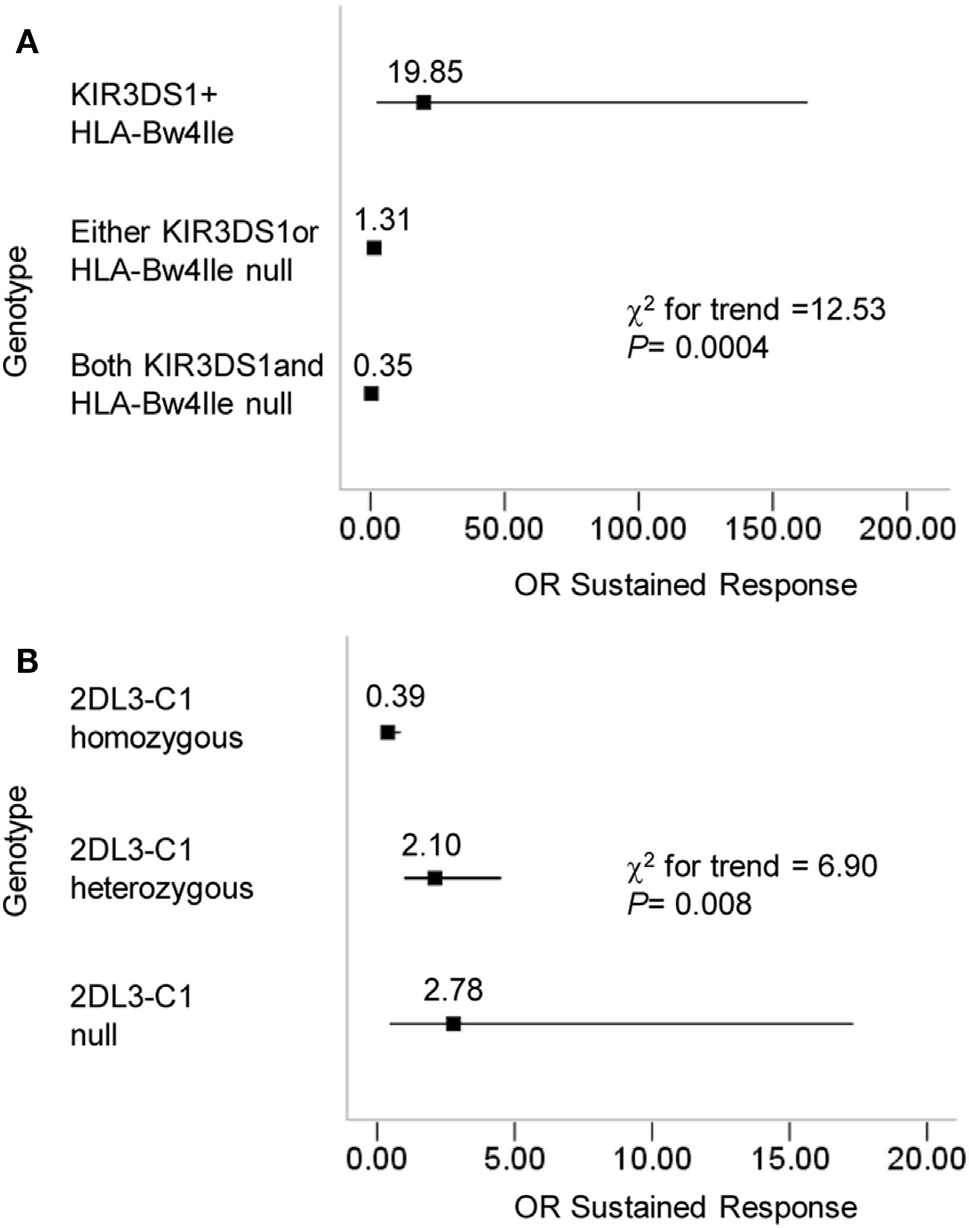
Progressive effects of KIR3DS1/HLA-B Bw4-80Ile and KIR2DL3/HLA-C1 on the outcome of chronic hepatitis B (CHB) therapy. **(A)** CHB patients (*N* = 119) were divided according to their KIR3DS1 and HLA-B Bw4-80Ile genotype into three categories: KIR3DS1/HLA-B Bw4-80Ile were both present; either the KIR3DS1 gene or the HLA-B Bw-80Ile gene was absent; both the KIR3DS1 and HLA-B Bw4-80Ile genes were absent. The chi-square test results for the trend were based on these three categories. **(B)** CHB patients were divided according to their KIR2DL3 and HLA-C genotypes. 2DL3-C1 homozygous individuals had the genotype 2DL3/2DL3-C1C1; 2DL3-C1 heterozygous individuals had the genotypes 2DL3/2DL3-C1C2, 2DL3/2DL2-C1C1, or 2DL3/2DL2-C1C2; 2DL3-C1 null individuals constituted the remainder and were missing KIR2DL3, HLA-C1, or both. The ORs and the 95% confidence intervals for the response to CHB treatment were calculated from 2 × 2 contingency tables. The results of a chi-square test for the trend were based on the three categories above. Abbreviations: OR, odds ratio.

Natural killer cells express distinct combinations of KIR genes in a stochastic manner (29). A previous study reported that the dose effects of KIR2DL3 and HLA-C1 genes influenced the resolution of HCV infection (16). In this study, we attempted to investigate the effect of a missing receptor or ligand based on the conclusion that the pairing of KIR3DS1/HLA-B Bw4-80Ile improves the response to therapy for CHB infection. To this end, we compared the OR values in three receptor–ligand pairing situations for KIR3DS1 and HLA-B Bw4-80Ile: (1) both present, (2) missing either KIR3DS1 or HLA-B Bw4-80Ile, and (3) both missing. Individuals who possessed the paired KIR3DS1 and HLA-B Bw4-80Ile le showed the greatest advantage in response to therapy for CHB infection in contrast to those lacking either the KIR3DS1 or the HLA-B Bw4-80Ile, or both (Figure 3A). We also observed a linear trend between the KIR3DS1/HLA-B Bw4-80Ile pairing and the odds of achieving a treatment response (χ^2^ for trend = 12.53; *P* = 0.0004) (Figure 3A). Beyond expectations, the pairing of KIR2DL3 and HLA-C1 correlated with the failure to respond to treatment (χ^2^ for trend = 6.90; *P* = 0.008) (Figure 3B).

### The KIR3DS1 and HLA-B Bw4-80Ile Genotype Predicts SR to Therapy among CHB Patients

Multiple logistic regression analysis was used to identify predictors of SR. A univariate analysis model constructed in this study verified a protective effect of KIR3DS1 in combination with HLA-B Bw4-80Ile to achieve SR for HBeAg-positive CHB patients treated with IFN-α (OR = 16.98, *P* = 0.01) (Table 4). Conversely, the KIR2DL3/HLA-C1 genotype was suggested as detrimental to SR achievement (OR = 0.37, *P* = 0.03) (Table 4). In addition, the haplotype and KIR2DS1/HLA-C2C2 genotype appeared to correlate less with therapy outcome (Table 4). These results suggested that KIR3DS1/HLA-B Bw4-80Ile, as the only genotype that was statistical significantly correlated with an improved outcome in the univariate analysis, can be used as an effective predictor for identifying the response to IFN-α therapy before treatment for HBV virus infection. The KIR/HLA correlations were not affected by age or gender, which showed no significant differences between groups when analyzed as conditioning variable (Table 4).

**Table 4:** Logistic regression model to predict achieving sustained response to therapy.

Variable	Code	β	SE	*P*-value	OR	95% CI
KIR3DS1-B Bw4I (KIR3DS1-B Bw4II/IT)	0: absent	2.83	1.15	0.01	16.98	1.80–160.56
	1: present					
KIR2DL3/2DL3-C1C1	0: absent	−0.98	0.45	0.03	0.37	0.16–0.90
	1: present					
KIR2DS1-C2C2	0: absent	1.96	1.23	0.11	7.11	0.64–79.13
	1: present					
Haplotype	0: AB/BB	−0.28	0.46	0.55	0.76	0.31–1.86
	1: AA					
**Conditioning variables**						
Age	—	–	–	n.s.	–	–
Gender	0: female	–	–	n.s.	–	–
	1: male					

*Gender and age were not included in the final model after the stepwise procedure*.

*KIR, killer immunoglobulin-like receptor; CI, confidence interval; n.s., not significant; OR, odds ratio; SE, standard error*.

*—, continuous variable*.

## Discussion

The prevalence of HBV infection is much lower in developed countries than in less developed ones (30, 31). On the one hand, social factors such as the large cardinal number of HBV carriers, poor health/medical conditions, and weak protection consciousness are considered nonnegligible factors associated with increased cases of HBV infection in less developed countries (32). On the other hand, we would like to think that the diversity of the immune system genes in various ethnic groups living at distinct regions as a result of evolution may lead to different outcomes for the same infection. Our study showed that all of the activating KIRs in the B haplotype were less prevalent in the Han Chinese (especially in CHB patients who were Han Chinese) than in Caucasians, including both European and US Caucasians. It is reasonable to speculate that there is a link between one or more activating KIRs in the B haplotype and hepatitis B infection.

Immunological factors have been reported to determine the disease outcome in CHB patients treated with IFN-α or IFN-α in combination with NAs (33–36). However, immunological factors are not ideal predictors due to their hysteresis. It is preferable to be able to predict pretreatment therapeutic effects. Host genotyping studies have demonstrated that polymorphisms of the HLA-DP genes are correlated with persistent chronic HBV infection in Han Chinese, indicating that host genes might influence the pathogenic processes (37). In the present study, a Han Chinese cohort of CHB patients enabled a comparison between patients who achieved SR to IFN-α treatment and those who exhibited NR. The genetic correlation analyses in this study indicated that CHB patients possessing the KIR3DS1 and HLA-B Bw4-80Ile allele genotype showed better therapeutic responses. We detected a trend toward pairing KIR3DS1/HLA-B Bw4-80Ile excess in CHB patients achieving a sustained response versus those with NR, although no difference was detected between CHB patients and healthy subjects. Moreover, it has been reported that the KIR3DS1/HLA-B Bw4-80Ile genotype showed no significant differences between CHB patients and those whose primary infections resolved spontaneously (18). Overall, the KIR3DS1/HLA-B Bw4-80Ile gene combination may predict success in achieving SR with IFN-α treatment but displays no correlation without treatment.

Previous studies have offered some evidence, mostly at the genetic level, for a potential role of activating KIRs in viral infectious diseases, such as those caused by HIV, HCV, and cytomegalovirus (38). The first evidence for a role of KIR3DS1 in viral infections came from genetic studies in HIV-infected individuals, indicating that an epistatic interaction between KIR3DS1 and HLA-B Bw4-80Ile delayed progression to acquired immunodeficiency syndrome (39). Additionally, evidence for the protective effect of KIR3DS1 in HIV infection was further strengthened by results from an *in vitro* study (40). Furthermore, activating KIR genes also played roles in affecting the HCV infection outcomes. KIR3DS1 and HLA-B Bw4-Ile80 have been confirmed to protect chronic HCV patients from developing hepatocellular carcinoma (41). The KIR2DS3/HLA-C2 genotype also played a role in affecting the outcome of HCV infection (42). Taken together, the evidence provided by these previous studies strongly supports our conclusion that activating KIRs, especially the KIR3DS1/HLA-B Bw4-80Ile combination, enhance antiviral immunity in HBV infection.

Two KIR2D/HLA-C genotypes influenced the outcome of a treatment response in chronic HBV infection. The KIR2DS1/HLA-C2C2 gene was more highly expressed in CHB patients of the SR group than in those of the NR group but did not reach a significant difference in the multi-logistic regression model. This lack of an effect may be attributable to the relatively small groups of subjects in the present study. KIR2DL3/HLA-C1 homozygosis seems to predict poor therapy outcome in this HBV model. Moreover, although the KIR2DL5 gene was more common in patients in the SR group than in the NR group, it is difficult to identify the role of this gene because KIR2DL5 is an orphan receptor. Similarly, HLA-Bw6 seems to be associated with a poor prognosis for HBV infection because it was more prevalent in patients in the NR group than in those in the SR group. However, there was no evidence that demonstrated that HLA-Bw6 can interact with KIRs.

It has been reported that HBV genotypes (A–H) show different geographical distribution, and are related to disease progression, response to antiviral therapy, and prognosis (43). The HBV genotypes of the CHB patients in our study are B and C genotypes which are predominant in Asian patients. The KIR/HLA associations were not affected by HBV genotype as a result of no significant differences between SR and NR groups when analyzed as conditioning variables in the multi-logistical regression model (partial data, data not shown). Considering the small number of patients and relatively simple genotypes (B and C), it is difficult to draw conclusions about the role of HBV genotypes in this study.

The most exciting study finding was our confirmation of the role of the KIR3DS1 and HLA-B Bw4-80Ile genotypes in HBV infection and resolution, particularly our observation of this effect for the first time in therapy for chronic HBV. KIR3DL1 recognizes HLA-B allotypes which contain Bw4 epitope spanning residues 77–83 (44, 45). Bw4-80Ile allotypes, which used to be considered as a preferential ligand over Bw4-80Thr allotypes, were not shown categorically stronger KIR3DL1 recognition than Bw4-80Thr in recent study (46). Moreover, KIR3DL1 polymorphisms were also shown to impact distinct HLA-I allomorphs recognition (46). A few HLA-A allotypes are also known to react with anti-Bw4 antibodies, but their role as a ligand for KIR3DL1 is controversial (47–49). The extracellular domains of KIR3DS1 and KIR3DL1 are extremely similar even though the receptor–ligand interaction between HLA-B Bw4-80Ile and KIR3DS1 has not been confirmed. Nevertheless, it has been observed that KIR3DS1 triggers cytolysis and IFN-γ production *in vitro* and enhances NK cell function in early HIV-1 infection (50). It is reasonable to speculate in such a model that the pairing of KIR3DS1 and HLA-B Bw4-80Ile most likely enhances NK cell activation based on the immunomodulatory effects of IFN-α. As such, CHB patients who possess the KIR3DS1/HLA-B Bw4-80Ile genotype have a greater potential to break immunotolerance as a results of the activation of specific groups of NK cells, and then successfully resolve HBV infection. Alternatively, the interaction with HLA-B Bw4-80Ile may impede the KIR3DL1-mediated inhibitory activity, thus causing the inhibition to be more easily overridden by the activation signal. Moreover, this effect was observed in both the HLA-B Bw4-80Ile heterozygous and homozygous CHB patients in the context of the KIR3DS1 gene and thus seems to be gene dose-independent. Activating KIR may also be involved in the process of NK cell licensing and it has been shown that KIR3DS1 positive NK cells in individual expressing HLA-B Bw4-80Ile alleles probably behave in hyporesponsive manner in order to maintain immune homeostasis under normal conditions (51). However, when non-physiological triggering signals such as HBV infections or IFN-α induced activation effects are given to cells in our study, the equilibrium that maintains tolerance could be disrupted and may become an important component of an efficient immune response. Analyses of individuals with KIR3DS1 and HLA-B Bw4-80Ile through sequential observation of HBV DNA, HBV serum markers, and a logistical regression model also suggested that neither KIR3DS1 nor HLA-B Bw4-80Ile alleles alone could account for the observed immune activation effect of the KIR3DS1/HLA-B Bw4-80Ile combination. A limitation of the present study is its relatively small sample size, thus leading to a large 95% confidence interval (CI) in some analyses and reduction of the statistical power. It is difficult to estimate the effects of some genes or gene combinations that did not reach statistical significance but revealed high ORs between groups in some comparisons.

Our data in the present study may contribute to the development of a relatively simple and specific host genotype test for predicting the outcome of chronic HBV patients receiving IFN-α therapy. However, considering the relatively low prevalence of the KIR3DS1/HLA-B Bw4-80Ile genotype, there are likely other mechanisms underlying NK cell activation in the absence of KIR3DS1/HLA-B Bw4-80Ile. Identifying such mechanisms will require further study. In summary, our findings support a role for activating KIR genes in the prevalence of HBV infection, extend the present understanding of the effect of the KIR3DS1/HLA-B Bw4-80Ile genotype on HBV infection, and advance our understanding of the mechanism of the immune responses in HBV clearance and drug responses.

## Ethics Statement

This study was approved by the ethics committee of the First Affiliated Hospital of Anhui Medical University (Grant No. K2010003). The clinical research was registered in the Chinese Clinical Trial Registry (Registration Number: ChiCTR-TRC-12002226).

## Author Contributions

Conceived and designed the experiments: HW, ZT, JL, and WL. Performed the experiments: WL, XS, BF, CG, and YL. Analyzed the data: HW and WL. Contributed reagents/materials/analysis tools: RS and JL. Wrote the paper: WL and HW. Obtained permission for use of clinical samples: JL and YY.

## Conflict of Interest Statement

The authors declare that the research was conducted in the absence of any commercial or financial relationships that could be construed as a potential conflict of interest. The reviewer UH and the handling editor declared their shared affiliation.

## Supplementary Material

Figure S1 Frequencies of KIR gene profiles identified in CHB patients.

Table S1 KIR genes frequencies amongst Han Chinese CHB patients, Han Chinese healthy subjects and US Caucasians (28).

Table S2 Baseline demographics and laboratory characteristics of the study cohort.

## Abbreviations

IFN-α, interferon alpha; HBV, hepatitis B virus; KIRs, killer-cell immunoglobulin-like receptors; NK cells, natural killer; HLA, human leukocyte antigen; CHB, chronic hepatitis B; HBeAg, hepatitis B e antigen; SR, sustained response; NR, no response; HBsAg, hepatitis B surface antigen; ALT, aminotransferase; NAs, nucleotide analogs; IFN-α, interferon alpha; HCV, hepatitis C virus; AIDS, acquired immunodeficiency syndrome; CI, confidence interval; OR, odds ratio.
