# Supplementary figures and images for: KIR3DS1/HLA-B Bw4-80Ile Genotype Is Correlated with the IFN-α Therapy Response in hepatitis B e antigen-Positive Chronic Hepatitis B
Source: Front Immunol. 2017 Oct 11;8:1285. doi: 10.3389/fimmu.2017.01285 (PMC5641573; doi:10.3389/fimmu.2017.01285)

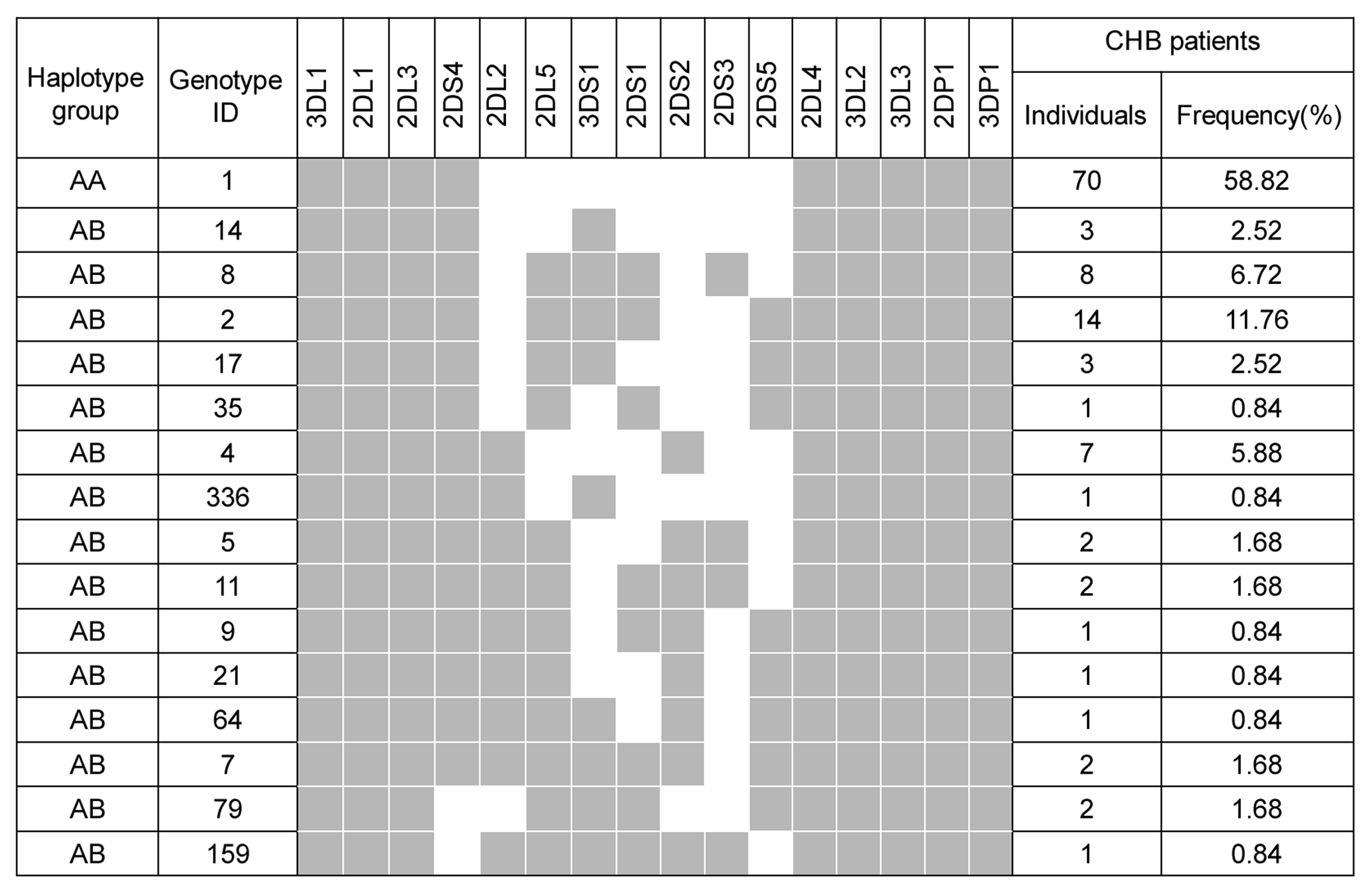

Supplement: Figure S1 — Frequencies of KIR gene profiles identified in CHB patients. [file Image_1.TIF]
