# Supplementary material for: KIR3DS1/HLA-B Bw4-80Ile Genotype Is Correlated with the IFN-α Therapy Response in hepatitis B e antigen-Positive Chronic Hepatitis B
Source: Front Immunol. 2017 Oct 11;8:1285. doi: 10.3389/fimmu.2017.01285 (PMC5641573; doi:10.3389/fimmu.2017.01285)
Supplement: Table S1 — KIR genes frequencies amongst Han Chinese CHB patients, Han Chinese healthy subjects and US Caucasians (28). [file Table_1.DOCX]

**Supporting Table 1. KIR genes frequencies amongst Han Chinese CHB patients, Han Chinese healthy subjects and US Caucasians.**

| Genetic Factor | Han Chinese | | Comparison between  CHB patients and healthy subjects | | | US Caucasians  N=255  (%) |
| --- | --- | --- | --- | --- | --- | --- |
|  | CHB patients  N=119 N(%) | Healthy  subjects  N=96 N(%) |  |  |  |  |
|  |  |  | OR^a^ | 95%CI | *P*^b^ |  |
| KIR2DL1 | 119(100) | 96(100) | ... | ... | 1.00 | 95.3 |
| KIR2DL2 | 15(12.6) | 15(15.6) | 0.78 | 0.36 - 1.69 | 0.92 | 52.9 |
| KIR2DL3 | 119(100) | 96(100) | ... | ... | 1.00 | 86.7 |
| KIR2DL4 | 119(100) | 96(100) | ... | ... | … | 100 |
| KIR2DL5 | 38(31.9) | 30(31.3) | 1.03 | 0.58 - 1.84 | 0.92 | 54.9 |
| KIR3DL1 | 119(100) | 94(97.9) | ... | ... | 1.00 | 94.9 |
| KIR3DL2 | 119(100) | 96(100) | ... | ... | … | 100 |
| KIR3DL3 | 119(100) | 96(100) | ... | ... | … | 100 |
| KIR2DS1 | 31(26.1) | 35(36.5) | 0.61 | 0.34 - 1.10 | 0.10 | 41.2 |
| KIR2DS2 | 17(14.3) | 15(15.6) | 0.90 | 0.42 - 1.91 | 0.78 | 53.3 |
| KIR3DS1 | 36(30.3) | 32(33.3) | 0.87 | 0.49 - 1.54 | 0.63 | 38.8 |
| KIR2DS3 | 14(11.8) | 13(13.5) | 0.78 | 0.35 - 1.73 | 0.70 | 29.4 |
| KIR2DS4 | 116(97.5) | 93(96.9) | 1.25 | 0.25 - 6.32 | 1.00 | 94.4 |
| KIR2DS5 | 23(19.3) | 25(26.0) | 0.68 | 0.36 - 1.30 | 0.24 | 34.5 |

Abbreviation: KIR, killer immunoglobulin-like receptor; HLA, human leukocyte antigen; CHB, chronic hepatitis B; OR, odds ratio.

KIR frequencies of US Caucasians were reported by Hollenbach JA et al.

OR^a^ > 1 indicates a protective association with response to treatment.

*P*^b^ values were calculated by using a Chi-square test from a 2×2 contingency table.

Statistically significant (*P*<0.05).
