# Supplementary material for: KIR3DS1/HLA-B Bw4-80Ile Genotype Is Correlated with the IFN-α Therapy Response in hepatitis B e antigen-Positive Chronic Hepatitis B
Source: Front Immunol. 2017 Oct 11;8:1285. doi: 10.3389/fimmu.2017.01285 (PMC5641573; doi:10.3389/fimmu.2017.01285)
Supplement: Table S2 — Baseline demographics and laboratory characteristics of the study cohort. [file Table_2.DOCX]

**Supporting Table 2.** **Baseline Demographics and Laboratory Characteristics of the Study Cohorts.**

**A.**

| Characteristics | CHB patients | Healthy subjects |
| --- | --- | --- |
| Age (yrs) | 28.64 | 37.63 |
| Gender (% male) | 72.27 | 52.08 |

**B.**

| Characteristics | CHB patients | | |
| --- | --- | --- | --- |
|  | SR | NR | *P* Value |
| Demographics | | | |
| Age (yrs) | 28.60(4.09) | 28.66(6.47) | 0.04 |
| Gender (% male) | 69.77 | 73.68 | 0.23 |
| Route (%): | | | |
| Horizontal transmission | 11(64.71) | 49(63.16) | 1.00 |
| Vertical transmission | 6(35.29) | 25(34.21) |  |
| Unknown | 20(46.5) | 2(2.63) |  |
| Treatment | | | |
| Peg-αIFN monotherapy | 10(23.3) | 20(26.3) | 0.62 |
| Peg-αIFN and adefovir combined therapy | 33(76.7) | 56(73.7) |  |
| Laboratory results (pre-treatment) | | | |
| Mean (SD) ALT, IU/mL | 217.7(127.2) | 219.1(120.6) | 0.55 |
| Mean (SD) HBV DNA, log c/mL | 6.9(1.5) | 7.5(1.3) | 0.38 |
| Mean (SD) HBsAg, log IU/mL | 3.7(0.8) | 4.0(0.6) | 0.01 |
| Mean (SD) HBeAg, log COI | 2.0(1.0) | 2.5(0.8) | 0.01 |
| Race: | | | |
| Han Chinese | 100% | 100% | 1.00 |

Abbreviation: SR, Sustained response; NR, No response.
