# Supplementary material for: KIR3DS1/HLA-B Bw4-80Ile Genotype Is Correlated with the IFN-α Therapy Response in hepatitis B e antigen-Positive Chronic Hepatitis B
Source: Front Immunol. 2017 Oct 11;8:1285. doi: 10.3389/fimmu.2017.01285 (PMC5641573; doi:10.3389/fimmu.2017.01285)
Supplement: Supplementary file 4 [file Data_Sheet_1.DOCX]

**Supporting Information**

**Supporting Figure 1. Frequencies of KIR gene profiles identified in CHB patients.**

**Supporting Table 1. KIR genes frequencies amongst Han Chinese CHB patients, Han Chinese healthy subjects and US Caucasians (**[**Hollenbach *et al.*, 2010**](#_ENREF_1)**).**

**Supporting Table 2. Baseline Demographics and Laboratory Characteristics of the Study Cohort.**

[1] J.A. Hollenbach, A. Meenagh, C. Sleator, C. Alaez, M. Bengoche, A. Canossi, G. Contreras, L. Creary, I. Evseeva, C. Gorodezky, R.A. Hardie, T. Hemming Karlsen, B. Lie, M. Luo, M. Martinetti, C. Navarette, D.C.M. De Oliveira, G. Ozzella, A. Pasi, E. Pavlova, S. Pinto, L.C. Porto, P. Santos, A. Slavcev, D. Srinak, S. Tavoularis, S. Tonks, E. Trachtenberg, S. Vejbaesya, and D. Middleton (2010). Report from the killer immunoglobulin-like receptor (KIR) anthropology component of the 15th International Histocompatibility Workshop: worldwide variation in the KIR loci and further evidence for the co-evolution of KIR and HLA. *Tissue Antigens* 76, 9-17.
